# Age-Dependence of the Peripheral Defocus of the Isolated Human Crystalline Lens

**DOI:** 10.1167/iovs.62.3.15

**Published:** 2021-03-10

**Authors:** Bianca Maceo Heilman, Ashik Mohamed, Marco Ruggeri, Siobhan Williams, Arthur Ho, Jean-Marie Parel, Fabrice Manns

**Affiliations:** 1 Ophthalmic Biophysics Center, Bascom Palmer Eye Institute, University of Miami Miller School of Medicine, Miami, Florida, United States; 2 Department of Biomedical Engineering, University of Miami College of Engineering, Coral Gables, Florida, United States; 3 Ophthalmic Biophysics, LV Prasad Eye Institute, Hyderabad, Telangana, India; 4 Brien Holden Vision Institute, Sydney, New South Wales, Australia; 5 School of Optometry and Vision Science, University of New South Wales, Sydney, New South Wales, Australia

**Keywords:** aging, crystalline lens, laser ray tracing, peripheral defocus, optics, myopia

## Abstract

**Purpose:**

To characterize the peripheral defocus of isolated human crystalline lenses and its age dependence.

**Methods:**

Data were acquired on 116 isolated lenses from 99 human eyes (age range, 0.03–61 years; postmortem time, 40.1 ± 21.4 hours). Lenses were placed in a custom-built combined laser ray tracing and optical coherence tomography system that measures the slopes of rays refracted through the lens for on-axis and off-axis incidence angles. Ray slopes were measured by recording spot patterns as a function of axial position with an imaging sensor mounted on a positioning stage below the tissue chamber. Delivery angles ranged from –30° to +30° in 5° increments using a 6 mm × 6 mm raster scan with 0.5-mm spacing. Lens power at each angle was calculated by finding the axial position that minimizes the root-mean-square size of the spot pattern formed by the 49 central rays, corresponding to a 3-mm zone on-axis. The age dependence of the on-axis and off-axis optical power and the relative peripheral defocus (difference between off-axis and on-axis power) of lenses were quantified.

**Results:**

At all angles, lens power decreased significantly with age. Lens power increased with increasing delivery angle for all lenses, corresponding to a shift toward myopic peripheral defocus. There was a statistically significant decrease in the lens peripheral defocus with age.

**Conclusions:**

The isolated human lens power increases with increasing field angle. The lens relative peripheral defocus decreases with age, which may contribute to the age-related changes of ocular peripheral defocus during refractive development.

The human lens grows continuously throughout life.^1^^–^^3^ This growth produces changes in the dimensions, shape, and internal structure that impact the optics of the lens and the whole eye.^4^^–^^8^ In turn, these effects impact the central (on-axis) and peripheral (off-axis) optical performance of the lens and could play a role in the development of refractive error of the eye.^9^^–^^12^ For example, Rozema et al.^13^ recently demonstrated that myopia onset was preceded by an accelerated rate of decrease in lens power in school-age children.

Age-related changes in the optical performance of the lens off-axis are of particular interest. According to some of the current theories for myopia, hyperopic peripheral defocus is a factor in the elongation of the eye during myopia development.^12^^,^^14^^,^^15^ Because the development of refractive error coincides with the rapid growth phase of the lens that occurs in early childhood,^6^^,^^16^ it is reasonable to expect that the optics of the lens contribute to the changes in peripheral defocus of the whole eye.

Previous studies on the crystalline lens optics have focused on characterizing the on-axis (paraxial) power in vivo and its changes with accommodation and age^17^^–^^23^ and in vitro.^24^^–^^26^ In a recent study, the isolated chicken lens was shown to have dramatically less off-axis astigmatism than a glass lens with similar power but a homogeneous refractive index, suggesting that the low off-axis astigmatism is due to the internal refractive index of the crystalline lens.^27^ In a previous study, we quantified the relative peripheral defocus of in vitro non-human primate lenses using a custom-built combined laser ray tracing and optical coherence tomography (LRT-OCT) system that enables on-axis and off-axis measurement.^28^^,^^29^ The goal of the present study was to characterize the peripheral defocus of the isolated human crystalline lens and its age dependence.

## Methods

### Donor Tissue

The study was approved by the Institutional Ethics Committee of the LV Prasad Eye Institute, Hyderabad, India, and adhered to the tenets of the Declaration of Helsinki. Data were acquired on 116 isolated lenses from 99 human donor eyes (age range, 0.03–61 years; postmortem time, 40.1 ± 21.4 hours) obtained from the LV Prasad Eye Institute's Ramayamma International Eye Bank, after the corneas had been removed under sterile conditions for transplantation. The eye bank, in accordance with its practices and procedures, obtained consent from the donor families to enucleate eyes for the purpose of transplantation, therapy, medical research, or education.

The donor age, gender, time and cause of death, time of enucleation, and time of experiment were recorded. The iris was removed, and the lens was carefully extracted using a lens spoon after the zonules were cut with Vannas scissors.^6^ The lens was then blotted dry to remove any remaining vitreous and placed in the testing chamber of the LRT-OCT system. Lenses with noticeable cataracts and capsular or other damage were discarded and not included in this study. Lenses from both eyes of a donor were included in the statistical analysis when available. A linear mixed-effects model analysis (described in more detail below) was used to account for the presence of both paired and unpaired samples.

### LRT-OCT System

**Figure 1.**
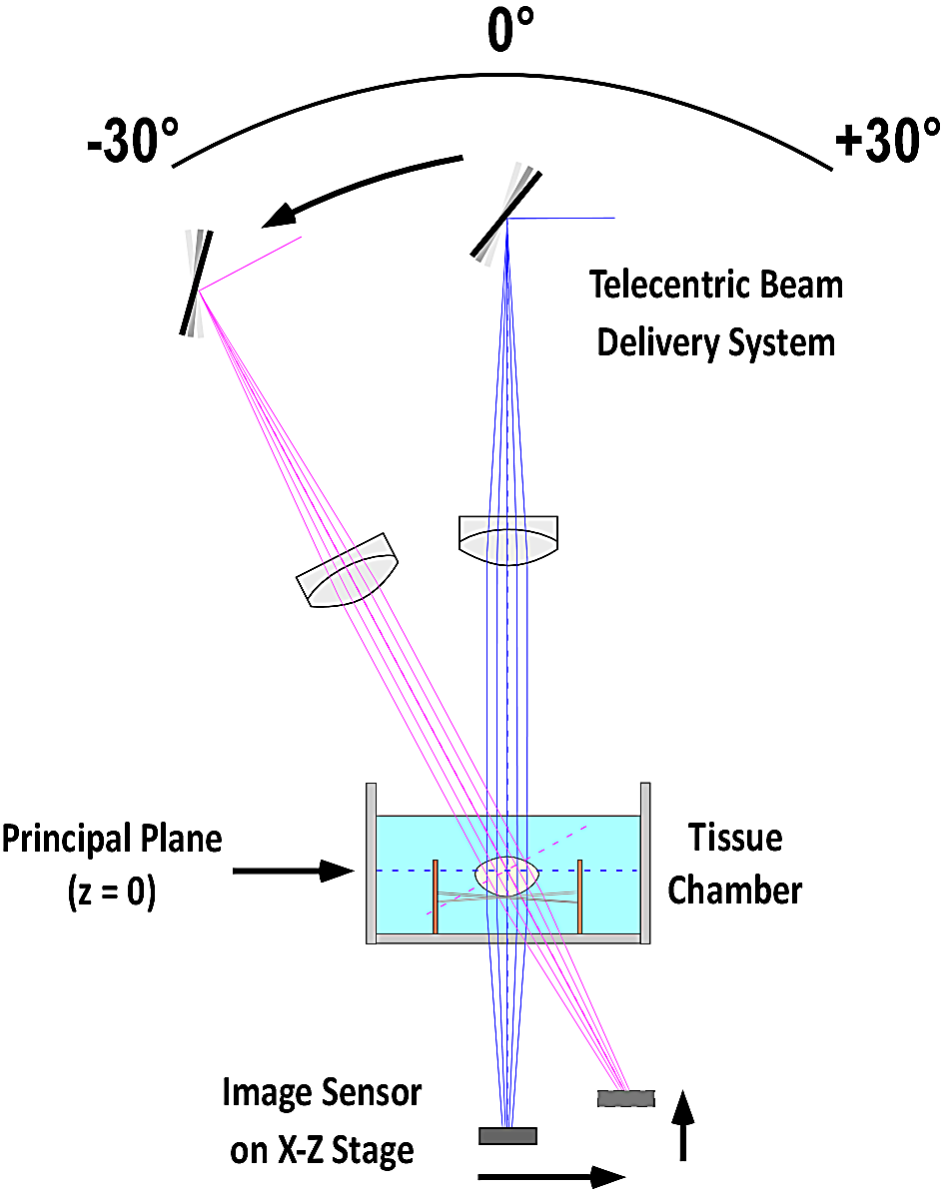
Schematic of the LRT-OCT system depicting the principle of off-axis ray trace data acquisition. The delivery probe rotates about the crystalline lens, and an image sensor mounted on a two-dimensional motorized positioning stage located below the tissue chamber is used to record the spot positions along each individual ray for all delivery angles. The principal plane was used as the reference position (*z* = 0) to calculate the distance *z_focus_*. The principal plane is shown as the *dotted blue line* at 0° and the *dotted magenta line* at –30°.

Experiments were performed on isolated human lenses using the custom-built LRT-OCT system to measure the on-axis and off-axis lens power (Fig. 1). A detailed description of the LRT-OCT system including an evaluation of its measurement precision and accuracy can be found in a previous publication.^28^ The following description summarizes the key aspects relevant to the present study.

The LRT system scans a narrow beam across the crystalline lens and records the slopes of the rays after transmission through the lens at each incident position of the raster scan. The system uses a custom-built scanning telecentric beam delivery system that produces a focused beam with a 53-μm spot diameter at the beam waist and a depth of focus (twice the Rayleigh range in air) of 5.1 mm onto the lens. The custom delivery probe is interfaced to a commercial OCT system operating at 880 nm (ENVISU R4400; Leica Microsystems NC, Inc., Morrisville, NC, USA). The custom delivery probe is mounted on a motorized rotation stage (T-RS60A; Zaber Technologies, Vancouver, BC, Canada) that pivots around the crystalline lens to allow acquisition of off-axis measurements. To acquire the ray slope data, an imaging sensor (DCC1545M-GL; Thorlabs, Inc., Newton, NJ, USA) is mounted below the tissue chamber on a two-dimensional (*x*, *z*) motorized positioning stage (T-LSR150B and T-LSR075B; Zaber Technologies). The image sensor has an active area of 6.66 mm × 5.32 mm and pixel size of 5.2 μm × 5.2 μm. The image sensor is used to record spots, corresponding to the cross-section of the laser beam, at multiple heights starting with the image sensor positioned approximately 2 mm below the tissue chamber. The position of the centroids of the spots are used to calculate the slope of each ray exiting the lens, thus enabling calculation of the lens power. The LRT data acquisition is fully automated via custom programming in LabView software (National Instruments Corporation, Austin, TX, USA).

During an experiment, the isolated lens is placed in a custom lens holder. The lens holder contains sutures arranged in a crisscross orientation, and the lens is gently positioned in the center of the sutures using a lens spoon. The lens holder is placed inside the tissue chamber, which rests on an aluminum holder mounted on a three-axis translation stage, allowing for precise centration and axial positioning of the crystalline lens within the LRT-OCT system. The tissue chamber was filled with balanced salt solution (BSS; Alcon, Inc., Fort Worth, TX, USA) in all experiments to ensure that the lenses were fully submersed and remained hydrated throughout the duration of the experiments.

### Experimental Procedure

The isolated lens was properly aligned and centered in the LRT-OCT system using real-time OCT images. The alignment protocol is described in detail in Ruggeri et al.^28^ The LRT system was programmed to automatically perform a 6 mm × 6 mm raster scan on the lens by sequentially delivering 169 rays with 0.5-mm spacing. Spot images were acquired along the optical axis for vertical positions from 1 mm to 8 mm below the tissue chamber with an axial (height) increment of 1 mm. LRT data were acquired at delivery angles ranging from –30° to +30° in 5° increments. The precision of the angle delivery was estimated to be less than 0.2°.

### Data Analysis

A custom MATLAB program (R2016b; MathWorks, Natick, MA, USA) was developed to calculate the centroid of each spot for all of the LRT images. The centroid position was used as an estimate for the *x,* *y* coordinates of the ray incident on the imaging sensor. For each individual ray, the coordinates of the spots acquired at the eight axial positions were used to calculate the ray slope in the *x* and *y* directions by performing a linear regression of the *x* and *y* coordinates of the spots. This analysis produced a map of ray slopes measured in air. The rays exiting the lens were reconstructed using these ray slopes, the incident ray position, and a correction for refraction through the window separating the medium and air.

The ray with zero slope at 0° incidence was used as the center ray for the root-mean-square (RMS) calculation. The 49 rays corresponding to the central 3-mm zone were used to solve for the position where the RMS spot size for the reconstructed rays was minimized. This position was used as an estimate of the best focus, *z_focus_*. The lens power was calculated using the formula 1.341/*z_focus_* to determine the on-axis and off-axis power for each lens, where 1.341 is the group refractive index of the hydration media at 880 nm.^28^ The distance *z_focus_* is measured relative to the image principal plane; therefore, the measured power corresponds to the effective lens power.^28^^,^^34^ The change in lens power or relative peripheral defocus, Δ*P*, was calculated as the difference between power at the respective delivery angle *P*(α) and the on-axis power *P*(0°), where Δ*P* = *P*(α) – *P*(0°).

The statistical analysis was performed using Stata 14.2 (StataCorp, College Station, TX, USA). A linear mixed-effects model using maximum likelihood estimation with random intercepts at the levels of donor and delivery beam orientation (positive and negative angles) was used to estimate the lens power as a function of donor age and delivery angle.^30^ *P* < 0.05 was considered statistically significant.

## Results

**Figure 2.**
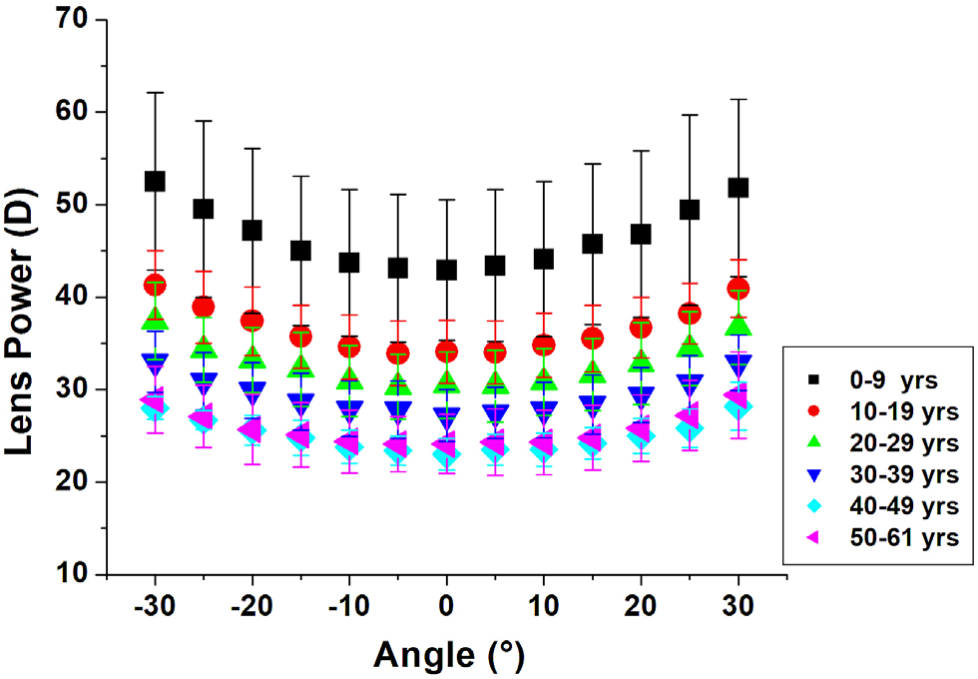
Lens power plotted with respect to delivery angle for isolated human lenses of varying ages. Following are the sample sizes per age group: 0 to 9 years, *n* = 18; 10 to 19 years, *n* = 15; 20 to 29 years, *n* = 25; 30 to 39 years, *n* = 34; 40 to 49 years, *n* = 12; and 50 to 61 years, *n* = 12. The average and SD were calculated using the linear mixed-effects model.

**Figure 3.**
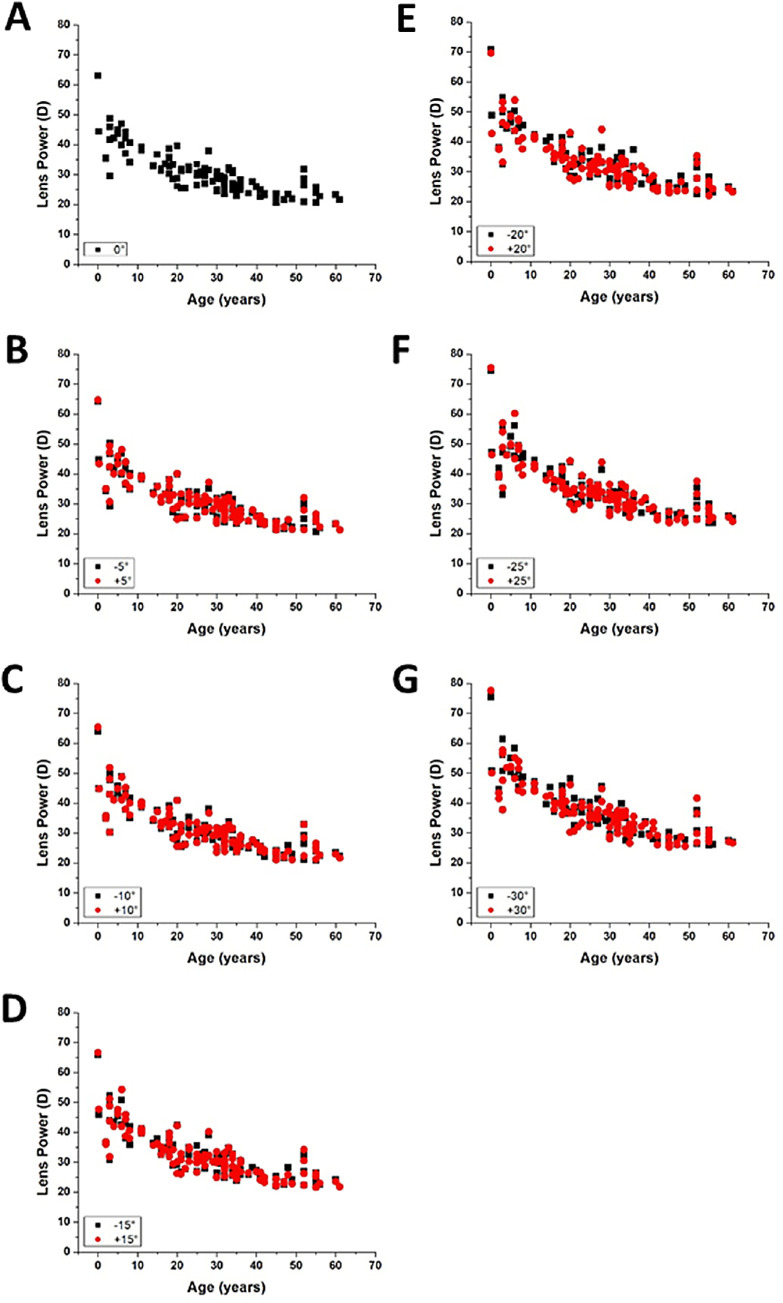
Lens power plotted with respect to age for delivery angles (**A**) 0°, (**B**) ±5°, (**C**) ±10°, (**D**) ±15°, (**E**) ±20°, (**F**) ±25°, and (**G**) ±30°. *P* < 0.0001 for all angles. The *P* values were calculated using the linear mixed-effects model.

Lens power increased with increasing delivery angle for all lenses in this study, corresponding to a shift toward more myopic defocus (Fig. 2). The profile of the lens power versus delivery angle curve is slightly steeper in the younger lenses. The power was found to decrease significantly with age at all delivery angles (Fig. 3).

Table 1 shows the lens power (average and SD) at all delivery angles grouped by age. As observed in Figure 2, the on-axis and off-axis lens power clearly decreased with age. On average, the on-axis power decreased by 18.9 D between the youngest (0–9 years) and oldest (50–61 years) age groups. The average off-axis power at 30° decreased by 22.5 D between the youngest and oldest age group.

**Table 1.** Lens Power (Average and SD) for Delivery Angles Ranging from –30° to +30° Grouped by Age

		Delivery Angle (°)												
Age (y)	Lens Power (D)	–30	–25	–20	–15	–10	–5	0	5	10	15	20	25	30
0–9 (*n* = 18)	Average	52.5	49.5	47.2	45.0	43.7	43.1	42.9	43.4	44.1	45.7	46.8	49.4	51.8
	SD	9.6	9.5	8.9	8.1	7.9	8.0	7.6	8.2	8.4	8.7	9.0	10.3	9.6
10–19 (*n* = 15)	Average	41.3	38.9	37.4	35.7	34.6	33.9	34.1	34.0	34.8	35.5	36.7	38.2	40.9
	SD	3.7	3.9	3.7	3.4	3.5	3.5	3.4	3.4	3.5	3.6	3.3	3.3	3.1
20–29 (*n* = 25)	Average	37.4	34.3	33.2	32.2	30.9	30.3	30.5	30.4	30.8	31.6	32.8	34.4	36.7
	SD	4.2	3.5	3.5	4.0	3.8	3.5	3.6	3.9	3.6	3.9	4.4	4.0	4.0
30–39 (*n* = 34)	Average	33.0	30.9	29.9	28.7	27.9	27.8	27.2	27.5	27.8	28.4	29.4	30.8	32.9
	SD	3.3	3.1	3.0	3.1	3.0	3.1	2.8	2.8	3.0	3.2	3.0	2.9	3.0
40–49 (*n* = 12)	Average	28.0	26.7	25.6	24.8	23.8	23.4	23.0	23.5	23.5	24.2	25.0	25.8	28.2
	SD	1.2	1.0	1.6	1.9	1.8	1.6	1.7	1.7	1.8	1.7	1.9	2.1	2.6
50–61 (*n* = 12)	Average	28.9	27.1	25.7	25.1	24.4	24.1	24.1	24.3	24.3	24.8	25.8	27.2	29.4
	SD	3.6	3.4	3.8	3.5	3.4	3.0	3.2	3.6	3.5	3.5	3.6	3.8	4.7

The average and SD values were calculated using the linear mixed-effects model.

**Figure 4.**
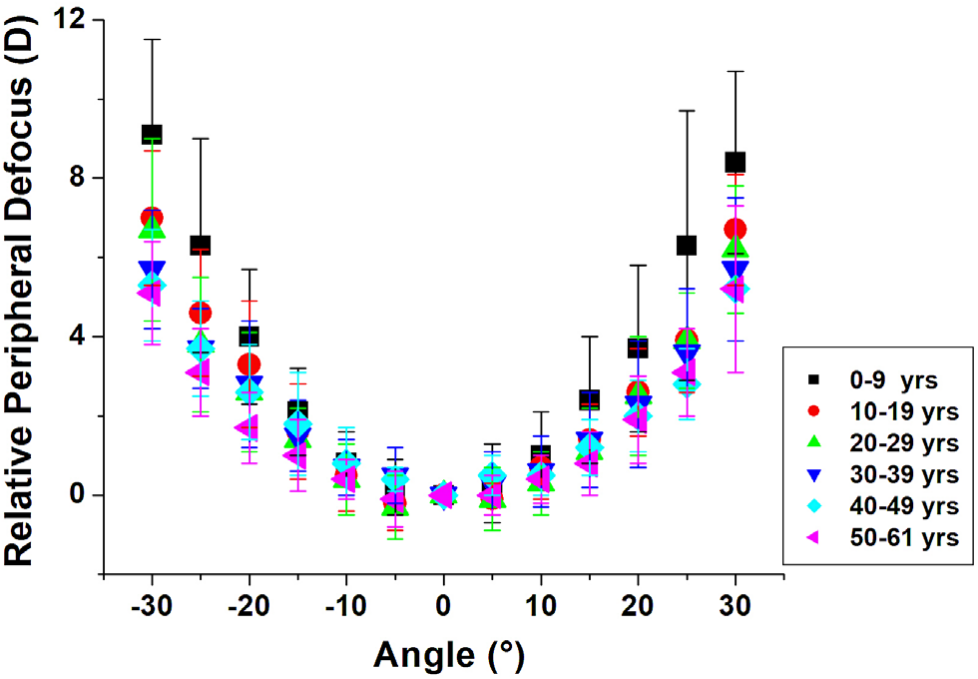
Lens relative peripheral defocus plotted with respect to delivery angle for isolated human lenses of varying ages. Following are the sample sizes per age group: 0 to 9 years, *n* = 18; 10 to 19 years, *n* = 15; 20 to 29 years, *n* = 25; 30 to 39 years, *n* = 34; 40 to 49 years, *n* = 12; and 50 to 61 years, *n* = 12. The average and SD were calculated using the linear mixed-effects model.

**Figure 5.**
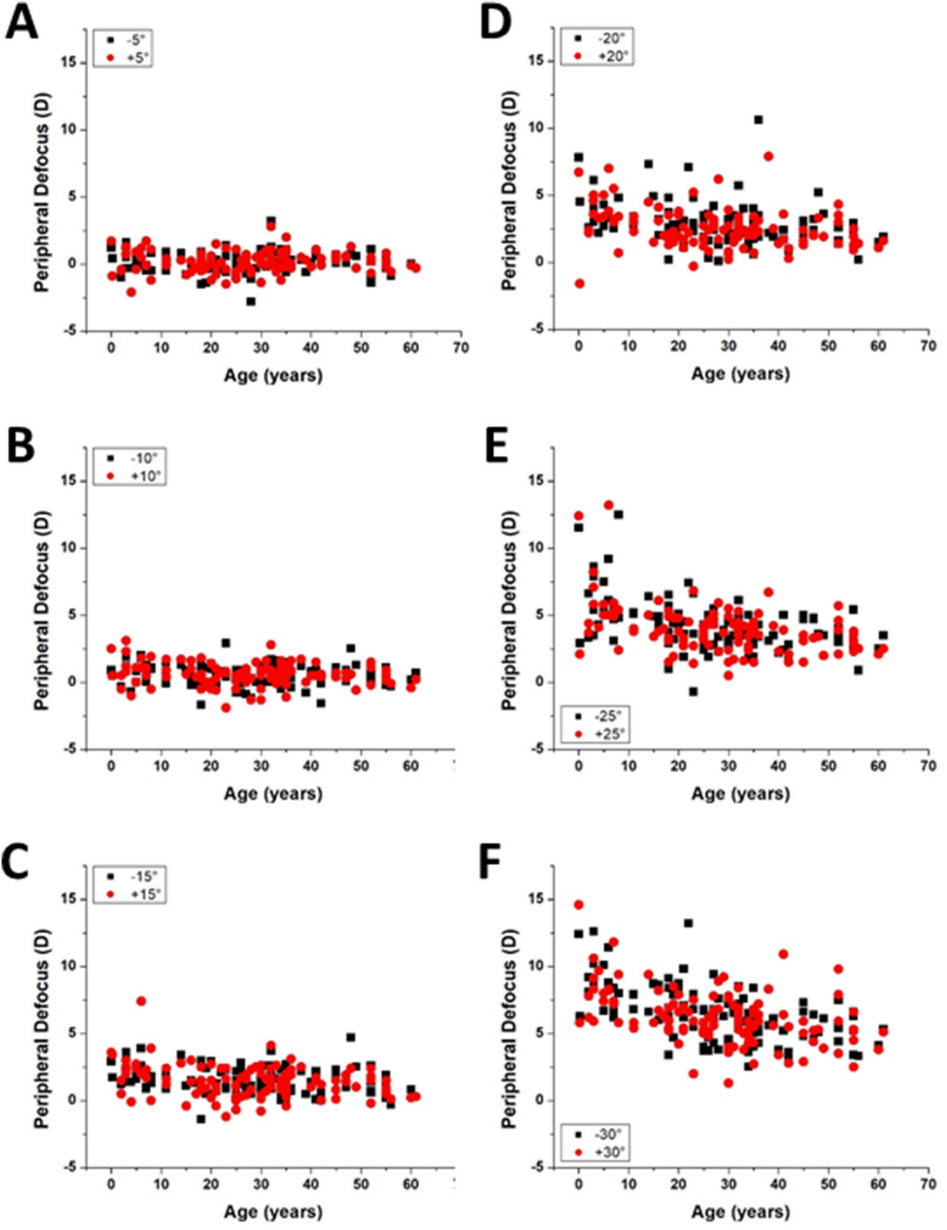
Lens peripheral defocus plotted with respect to age for incidence angles (**A**) ±5°, (**B**) ±10°, (**C**) ±15°, (**D**) ±20°, (**E**) ±25°, and (**F**) ±30°. *P* values: 0.74 at ±5°, 0.08 at ±10°, 0.0006 at ±15°, and <0.0001 at ±20°, ±25°, and ±30°. The *P* values were calculated using the linear mixed-effects model.

The profile of the relative peripheral defocus versus delivery angle curve appears to flatten with age (Fig. 4). The relative peripheral defocus of the isolated human lens was plotted with respect to delivery angles in Figure 5. At small angles (±5°and ±10°), the relative lens peripheral defocus was independent of age, within the precision of the measurements. However, at steeper angles (±15° to ±30°), the relative peripheral defocus decreased significantly with age.

Table 2 shows the relative peripheral defocus of the lens (average and SD) at all delivery angles grouped by age. As observed in Figure 4, the peripheral defocus of the lens increased with increasing delivery angle. Notably, at the steeper delivery angles, the peripheral defocus was greater in younger lenses than in older lenses. On average, the relative peripheral defocus at 30° decreased by 3.3 D between the youngest (0–9 years) and oldest (50–61 years) age groups. As a reference, Supplementary Table S1 shows the lens power for delivery angles ranging from −30° to +30° for the individual lenses in this study.

**Table 2.** Lens Relative Peripheral Defocus (Average and SD) for Delivery Angles Ranging from –30° to +30° Grouped by Age

		Delivery Angle (°)												
Age (y)	Lens Power (D)	–30	–25	–20	–15	–10	–5	0	5	10	15	20	25	30
0–9 (*n* = 18)	Average	9.1	6.3	4.0	2.1	0.8	0.2	0.0	0.3	1.0	2.4	3.7	6.3	8.4
	SD	2.4	2.7	1.7	1.1	0.8	0.7	0.0	1.0	1.1	1.6	2.1	3.4	2.3
10–19 (*n* = 15)	Average	7.0	4.6	3.3	1.6	0.5	–0.2	0.0	–0.1	0.7	1.4	2.6	3.9	6.7
	SD	1.7	1.6	1.6	1.2	0.9	0.7	0.0	0.4	0.8	0.9	1.1	1.3	1.4
20–29 (*n* = 25)	Average	6.7	3.8	2.6	1.4	0.4	–0.3	0.0	–0.1	0.3	1.1	2.5	3.9	6.2
	SD	2.3	1.7	1.5	0.8	0.9	0.8	0.0	0.8	0.8	1.1	1.5	1.2	1.6
30–39 (*n* = 34)	Average	5.7	3.7	2.8	1.5	0.7	0.5	0.0	0.3	0.6	1.4	2.3	3.6	5.7
	SD	1.5	1.0	1.6	0.9	0.7	0.7	0.0	0.8	0.9	1.2	1.6	1.6	1.8
40–49 (*n* = 12)	Average	5.3	3.7	2.6	1.8	0.8	0.4	0.0	0.5	0.5	1.2	2.0	2.8	5.2
	SD	1.4	1.2	1.2	1.3	0.9	0.3	0.0	0.5	0.5	0.7	0.9	0.9	2.1
50–61 (*n* = 12)	Average	5.1	3.1	1.7	1.0	0.4	–0.1	0.0	0.0	0.4	0.8	1.9	3.1	5.2
	SD	1.3	1.1	0.9	0.9	0.5	0.7	0.0	0.5	0.6	0.8	1.1	1.1	2.1

The average and SD values were calculated using the linear mixed-effects model.

## Discussion

This study presents measurements of the isolated human crystalline lens peripheral defocus and its age dependence using LRT. To the best of our knowledge, these results are the first measurements of the human lens relative peripheral defocus. The human lens was found to have a significant amount of peripheral defocus at all ages with an increase in power in the periphery, corresponding to a shift toward myopic defocus. This study also demonstrates that the relative peripheral defocus of the lens changes with age. These findings suggest that the human lens contributes significantly to the peripheral optical performance of the eye and that the continuous growth of the lens contributes to the age-related changes in the peripheral optical performance of the whole eye.

Our isolated lens power measurements are consistent with previous studies that quantified the power of the human lens (on-axis) and its changes with age.^13^^,^^25^^,^^31^ We performed a linear regression on the lens power versus age for young lenses (3–8 years), an age span that coincides with the rapid growth phase of the lens in early childhood. Our on-axis measurements (–0.79 D/y) are comparable to the in vivo measurements by Rozema et al.^13^ on Singaporean children who were new myopes (–0.71 D/y).

Our results are also in agreement with our previous study that quantified the relative peripheral defocus of cynomolgus monkey lenses mounted in a lens stretcher.^29^ Monkey lenses were found to have significant myopic peripheral defocus, which varied with stretching. In the present study, the human lenses were isolated so we cannot quantify the effect of stretching to simulate disaccommodation. Because the unstretched lens has no forces acting upon it, this state is analogous to the fully accommodated lens. Thus, we compared our results on isolated human lenses with the unstretched monkey lenses from the previous study. The monkey lenses had a significantly higher power than the human lens. The average on-axis power of the monkey lens was 52.0 ± 3.4 D in the unstretched state. At the +20° delivery angle, the relative peripheral defocus of the young monkey lens was significantly greater than that of young human lenses: on average, 11.0 D for monkey lenses (ages, 3.8–6.8 years) compared to 3.3 D for human lenses (ages, 0–9 years). These results suggest that the general peripheral power profiles of monkey and human lenses are similar, even though peripheral defocus is more pronounced in monkey lenses.

Furthermore, the study on monkey lenses^29^ showed that stretching the tissue radially by 5.25 mm produced an average change of 3.1 ± 2.1 D in peripheral defocus (at a 20° delivery angle). It is reasonable to expect that the peripheral defocus of the human lens would change less with stretching. Therefore, it is unlikely that stretching the human lens would completely compensate for the difference in relative peripheral defocus measured in the present study. In other words, we expect that the stretched lens, corresponding to relaxed accommodation, will also exhibit a significant relative peripheral defocus.

In addition, we also expect that the age dependence of the lens peripheral defocus will be less pronounced in the relaxed state than in the accommodated state, because the central lens power is less dependent on age in the relaxed state. Measurements on stretched lenses are required to determine if the peripheral defocus of the relaxed lens is age dependent. However, altogether, our results on the isolated human lens (which corresponds to the accommodated state in vivo) clearly demonstrate that there are significant changes in the lens peripheral defocus with age that may play a role in refractive development.

The ray trace experiments were performed on an isolated lens using parallel beams, whereas in the human eye the beams entering the eye would be focused by the cornea. As discussed in our previous publication on monkey lenses,^29^ we do not expect that the input ray vergence significantly affects the peripheral defocus. The present study also quantified the best focus of the lens on- and off- axis using the rays in the central 3-mm zone. We found that the ray slope varied linearly with input ray height in this zone, demonstrating that the contribution of aberrations is minimal in the central 3 mm of the lens. We therefore expect that the position of the RMS best focus calculated in our study corresponds to the paraxial focus.

The LRT-OCT system measures the peripheral defocus relative to a flat surface corresponding to the imaging sensor, whereas the retina is curved. On average, the radius of curvature of the tangential image field corresponding to the measured peripheral defocus was –28.7 ± 9.3 mm for the isolated human lenses in this study. The radius of curvature of the image field of the human lens is therefore significantly greater (flatter) than the typical radius of curvature of the human retina (roughly 12 mm).^32^^,^^33^ This observation suggests that the lens produces a hyperopic defocus relative to the retinal contour and that age-related changes in the lens contribute to increasing this hyperopic defocus.

In summary, our study demonstrates that the human crystalline lens power increases with increasing field angle. When retinal curvature is taken into account, the lens is found to contribute to hyperopic peripheral defocus. The relative peripheral defocus of the human lens was found to decrease with age, which could play a role in refractive error development by contributing to a progressive increase in hyperopic defocus with age.

## Supplementary Material

Supplement 1
